# The International Congress of Medicine

**Published:** 1913-08-16

**Authors:** 


### THE INTERNATIONAL CONGRESS OF MEDICINE.

### An Expert Synopsis of the Proceedings.

There are a good many scientific workers who do not hesitate to declare their belief that Congresses of all sorts are a waste of time and money. They say that nothing new is disclosed—or very seldom, at any rate—and that the "windbags" and self-advertisers use such meetings as opportunities for taking unto themselves credit which should go to the genuine research worker, whose brains are picked by men merely out for popular applause. There is a certain element of truth in this generalisation; but to the International Congress just ended in London it most decidedly does not apply. Not only from a popular point of view, but also from that of strict medical science, the right men were chosen for the big occasions; and the tremendous public interest which has been aroused merely reflects the excellent organisation of the Congress and the discretion of the Committee which took it in hand. The Congress as a whole has been an unqualified success, and if every scientific Congress were as well managed there would be few who would support the opponents of these gatherings.

### Scientific Stocktaking.

It would be idle, however, to pretend that every speaker in every section contributed usefully to the proceedings; or that the advertising pseudo-scientist was completely suppressed. But this does not alter the fact that the opportunities of this type of medical man were firmly, yet tactfully, curtailed as much as possible; and that in general an extraordinarily high level of scientific medicine was maintained. Again, it is true that very few new facts emerged, and that the great majority of the "reporters" of the twenty-six sections and sub-sections merely recapitulated the researches and advances of the last few years. But this very repetition, irritating as it may be to the specialist who is fully up to date in his own subject and mainly anxious for fresh worlds to be conquered, really constitutes a valuable feature of an International Congress. For by this means the general practitioner, and the specialist, too, have a chance of taking stock of progress, of interchanging views with men of other nationalities, and of surveying the field preparatory to fresh campaigns against disease. Taking a broad view of the whole of the proceedings we feel justified in saying that the Congress was not only an unprecedented success from the point of view of the doctors, both scientifically and socially, but was equally momentous from that of the general community, whose members, if they did not see most of the game, saw at least enough to interest them profoundly as onlookers with a personal stake in the issues.

### The General Addresses.

The leading personalities among our visitors were briefly mentioned in our columns last week (p. 549), and are by this time familiar to those who have followed the discussions and papers, at any rate by name. The "general addresses," of which there were five, naturally demand consideration first of all, and indeed they well deserve it on their own intrinsic merits. The introductory speech of Sir Thomas Barlow, the President of the Congress, on the opening day was well worthy of his high reputation in the medical world. It was mainly retrospective, and laudatory of the great men who took part in the International Congress of 1881, the last one held in London until this present year. Pasteur, Lister, Koch, and many others received their fitting eulogy. In a striking passage Sir Thomas Barlow compared those great names of 1881 to mountain peaks, with deep valleys between; whereas the present state of knowledge, based so largely upon their labours, more resembles a high tableland of much more uniform elevation. We are not quite sure that this simile will be thought in a few years' time to be accurate; for there are still notable men in the profession whose exploits are those of pioneers, and whose genius attains much higher flights than those of ordinary men. But we may admit that it does express a fundamental distinction between the state of medical science in 1881 and that of 1913.

### Chauffard, Cushing, and Ehrlich.

The general address in medicine was delivered by Professor Chauffard, of Paris, on the subject of prognosis. It was an extremely clever and well-thought-out piece of work, full of Gallic ingenuity; and well worthy of its author's high reputation and his position as general spokesman, as it were, of the French medical profession. Professor Harvey Cushing's address in surgery was even more to the taste of the Congress. It had been anticipated that this well-known American surgeon might deal especially with the surgery of brain tumours, the pituitary gland, and similar triumphs of recent chirurgical progress with which his name has been closely associated. Instead of doing so, Professor Cushing took much broader ground; and in so doing proved that his own particular bent towards cranial operations had not narrowed his outlook upon other aspects of the great science of surgery.

In a speech which takes rank among the very best and most eloquent delivered during the Congress he defended at great length the practice of vivisection, and exposed the dangers involved by the anti-vivisection agitation. This vigorous pronouncement was particularly opportune owing to the recent outburst of anti-vivisection activity, as signified by Miss Lind-af-Hageby's libel action and Sir F. Banbury's Bill for the exemption of dogs from vivisection. True, both these attempts to annoy and hinder vivisectionists failed utterly; but all the same, the spectacle of a leading trans-atlantic surgeon corroborating and confirming the views of British scientists has not been lost upon the public. Great credit is due to Professor

Cushing for his outspoken address, which is moreover of itself a very fine piece of logical exposition and scientific analysis.

Perhaps the next most important point made in this address, which was full of good things, was the emphasis laid by Professor Cushing on the value of inter-institutional visits. He was, of course, referring to the visits which many surgeons are now in the habit of paying to rival clinics, both in their own and in other countries, whereby advances in technique and in diagnosis are rapidly spread through the surgical profession instead of remaining the exclusive property of one particular school or one particular country. We are glad to say that this custom has been making great headway of late years among British surgeons, who were somewhat slow in adopting it at first, for it is a tendency in every way to be encouraged. Nor should it be confined to surgeons; other institutional officers, medical and lay, have much to gain by seeing and studying each other's methods. Certainly many of our foreign visitors have set us object-lessons in this matter, for they have been most assiduous in their visits to our London hospitals during the Congress week.

### A German Genius.

In pathology the general address was delivered by Professor Paul Ehrlich, who naturally enough took for his text the chemico-biological theories of immunity, infection, and toxæmia, which he has been working out for some years. Many people associate Ehrlich simply with the much-talked-of salvarsan, his sensational discovery of three years ago; and outside bacteriological circles this is perhaps his chief title to fame. It is well to realise, however, that Ehrlich is a man of most exceptional genius, whose bold speculations and imaginative theories have been the outstanding features of bacteriological progress any time this last twelve or fourteen years. Backed by masses of facts, patiently accumulated and ably arranged, Ehrlich's theories had won appreciation and acceptance all over the world long before the conception of his *therapia sterilisans magna* was given to the world, or salvarsan ("606") invented and put upon the market

In the general address, Professor Ehrlich dealt mainly with his theories, leaving the practical exposition of salvarsan treatment for a joint discussion of two other sections more nearly concerned with it than was that of pathology. He was at great pains to defend his "complete radical cure" idea of dealing with infective diseases. What he aims at—and had at first hoped to accomplish with salvarsan—is to find some substance so inimical to the micro-organisms and parasites of disease that a single injection of it shall kill every one of them existing in the patient. This could, as a matter of fact, probably be accomplished as things are; but only by such a dose of some powerful bactericide as would kill the patient too. Ehrlich's ideal is to find a drug which will kill off the parasites and yet do no harm to their host, the patient. If he, or anyone else, could discover a drug which would with certainty do this for a single disease, an enormous step in advance would have been taken. But he looks forward to more than this—that is, to producing a drug which shall so act in regard to a whole group of organisms, or even be fatal to every organism of every kind.

### The "Elixir of Life" Conception.

This startling conception, nothing less than the cure of all infective diseases by one dose of one wonder-working drug, has not yet been attained, needless to say. But Ehrlich believes he will attain it, or at least that it is attainable; and his accomplishments in the past have been so extraordinary that it would be unduly sceptical to deny altogether the possibility of his dream being realised. Salvarsan, it is now fairly well established, is efficacious against a good many infective diseases, though it has not the immediate complete radical curative effect which the German professor hoped it would have. Still, it is certainly the most potent drug in existence; and it behoves us to remember that in it synthetic chemistry has achieved a triumph over organic *materia medica* which would have been deemed totally impossible a few years ago. Now synthetic chemistry is an infant science at present, whereas *materia medica* is not; so there is no telling what the future may bring about. Abstruse as Professor Ehrlich's defence of his theories was, his audience thoroughly enjoyed his address, and he had a great reception. It will not be surprising if by the time of the next International Congress in London, Ehrlich's name has been promoted to the company of the immortals—Lister, Pasteur, and the rest. As a matter of interest we may note that the professor is nearly sixty; so that if he lives to see that Congress, and if it meets in London after a similar interval of time to that which elapsed between this just concluded one and its predecessor, he will be well over ninety years of age. We wish him length of years to visit us again when that time comes. *Prosit!*

### Professor Bateson on Heredity.

Here, too, the organisers of the Congress had been particularly happy in their selection of a scientist for the general address of the day (Monday last). Professor Bateson has not merely scientific credentials of the most irreproachable kind, and an originality which has already borne fruit in much valuable research, but has also that happy faculty of making abstruse things comprehensible to those whose acquaintance with his subject-matter is elementary. Mendel's teachings as he left them are not well adapted for popular comprehension. But Professor Bateson's exposition of the Mendelian doctrines, whose position he has himself done so much to defend and to strengthen, must have been acceptable to many who are desirous of comprehending the leading principles of the work and theories of the celebrated *abbé*, so long neglected but now on a pinnacle of biological fame. With the rapidly awakening interest of all classes of the community in problems of heredity as affecting the public well-being, it was particularly important that any public pronouncement on the question made at this Congress should be both authoritative, intelligible to the layman, and broad in conception. In all these respects the general address on heredity was quite admirable, and the thanks of the medical profession, no less than of educated citizens at large, are due to Professor Bateson for his illuminating essay.

What, of course, the public does not realise is that the true value of the Congress will not be realisable till several weeks, perhaps months, have passed. The public inevitably has had to be content with the barest skeleton of the ideas, theories, or practical points put forward, and the lay newspapers, though, no doubt, doing their best to present a kind of summary, have been unable to avoid the temptation to titivate public curiosity by the attempt to startle its credulity. We shall see, however, in the next few months that, led by the technical periodicals, certain questions will grow to the full stature of their importance, and then the effects and results of the Congress will percolate through to practical life.

### The Work of the Sections.

Turning now to the work of the individual sections, which (counting in sub-sections) totalled twenty-six, it is only possible to deal very briefly with a small fraction of the work which was got through. Broadly speaking, the most important papers and contributions were those which were presented in connection with the set debates on predetermined subjects, inaugurated each by some one of special authority on the question under review. Since each section met on six days, and there were sometimes more topics than one set down to be formally "reported" each day, it will be seen that a formidable mass of material is at the disposal of those who would follow the deliberations of the sections. It frequently happened that independent papers put down on topics selected by their authors were crowded out by the number of speakers in the regular debates; and in some cases it was necessary to hold supplementary sittings in separate rooms to enable those members interested in the former communications to have an opportunity of listening to them.

Curiously enough, the members of each section were quite convinced not only that their own section was the most important in the whole Congress, but also that greater progress had occurred in that particular specialty during the past few years than in any other in the same space of time. This sentiment was voiced in a good many sections, and pervaded also those in which it was not publicly expressed. We have no intention here of attempting to decide between these rival claims, and shall do no more than pick out a few of the most vital items with which the programmes of all the sections teemed.

### The History of Medicine.

As was expected, the papers presented to this section were of the most diverse kind. A lady, Miss Stawell, tried to prove that St. Luke, the "good physician," who wrote the Gospel of that name and also Acts, was not a Greek, as has usually been supposed, but a Roman. Whether this proposition was or was not sustained to the satisfaction of Testament critics we do not know; but at least we are certain that Miss Stawell's exposition of the general status of medicine in early Christian times, and of the conditions of medical practice in Rome, were of surpassing interest. Of equal fascination was Dr. Caton's account of the shrine and temple of Hippocrates at Cos, investigated lately under German auspices by Professor Hertzog, of Tübingen. His reconstruction of the temple and its precincts reveals a distinctly institutional type of building, for consulting rooms, waiting rooms, operating rooms, lecture rooms, dispensary, and library have been identified. What would one not give for a full account of the management of this ancient hospital, or for a vision of the operative and other treatment which the patients received! As wards are not mentioned, it may be presumed that an out-patient department alone existed, from which we may infer that this is the oldest branch of hospital construction and organisation.

### A New Type of Mental Hospital.

Another affair which excited considerable attention from the reporters for the lay press was the exhibition of paintings and drawings by patients in mental hospitals, arranged at Bethlem by Sir G. H. Savage. It is well known that quite a fair proportion of the insane employ the abundant leisure they enjoy in pictorial efforts. As a general rule these are crude to a degree, and are often more in the nature of caricatures lampooning the lunacy laws or persons against whom they harbour some special grievance. But in a certain number of cases great talent, even genius, is exhibited. Some of the exhibits at Bethlem were the work of a celebrated artist whose mental disorder had actually driven him to homicide. Others which attracted notice were some representatives of the cubist and post-impressionist schools; these, we fear, provoked cheap and obvious gibes from some of the visitors.

While dealing with this subject we must not overlook the very valuable discussion in the psychiatry section on the aims and results of psychiatric clinics. At Boston and Baltimore such institutions are in full swing, and the experience there gained and narrated to the section should prove of great value to the managers of the similar institution which London will possess in the Henry Maudsley Hospital, shortly to be erected. Put very briefly, the object of this new type of mental hospital is the prevention of mental breakdown, obviously better, if it is possible, than anything which mental hospitals can effect. By dealing with those in whom mental disorder is merely threatened, not actually present, the onset of madness can, in very many cases, be prevented. And another very important function of a psychiatric clinic is the education of medical practitioners in the symptoms, signs, and treatment of mental changes in the early and controllable stage.

### The Medical Conscience.

One of the outstanding features of the Congress has been the recognition, alike by the speakers, the public, and the press, of the right and duty of the medical profession to take a greater share now than in the past in the economical and political aspects of public health. This particular point was well brought out in Dean Inge's sermon in St. Paul's Cathedral on Sunday, August 10, the whole of which well repays study alike for its philosophical appreciation of the principles at stake and for their scholarly and dignified elaboration. In some European countries, notably in the Scandinavian kingdoms, recognition of these duties has been much more general than it has with us; but it is most satisfactory to see the arousing of the medical conscience which has certainly taken place as an outcome of the Congress meetings. In this connection particular stress may be laid on the joint debate held by the section of naval and military medicine with that of forensic medicine on the subject of syphilis. The speakers very wisely, as we had ventured in these columns to suggest beforehand that they should, refrained from entering into the highly controversial topics involved by those compulsory prophylactic measures of legislation which were abandoned many years ago. But the exchange of international opinion upon this matter turned out to be extremely valuable, and the session excited perhaps as much interest as any other throughout the Congress. Significantly, on the very same day as this debate, the Prime Minister announced in the House of Commons that an inquiry into this subject will be proposed by the Government. It is true Mr. Asquith avoided the words "Royal Commission," and is therefore not necessarily pledged to anything very much. But it may be that this announcement was in the nature of a feeler, and that he is quite willing to proceed to more thorough measures if the medical profession will push home the agitation commenced by Sir Malcolm Morris.

In several of the sections pronounced differences of opinion were evident over certain subjects, and were freely expressed. In the daily press the sporting character, if one may so term it, of these encounters between opposing scientists seems to have appealed to the editors, and through the press to the public. Such outspoken debates are a thoroughly healthy symptom of a scientific outlook; Science is no respecter of persons or doctrines, and so long as regard for truth is the ruling passion among her followers we may be sure that progress will continue. Animated as some of these discussions were, there was never anything but the best of feeling about them, and everyone took criticism in good part. Indeed, this has been one of the most noticeable features of the whole Congress, the unprecedented absence of international jealousy, of personalities, and of prejudices. Influential laymen who have attended the debates declare that the fresh atmosphere of earnestness, sincerity, and mutual courtesy that has prevailed at the gatherings has been a perfect revelation to them of the spirit which animates the scientific medical world. We rejoice that this should be so, and would fain believe that British honesty and open-mindedness have had something to do with it; and that the Congress in this way will leave a permanent mark upon the future of medicine. As an instance of this proper scepticism and outspokenness, we may quote especially the proceedings of the Section of Psychiatry, when the doctrines of Freund on psychoanalysis were attacked by Professor Janet in one of the most brilliant papers read during the Congress week, and defended by one of Freund's colleagues. There were several other instances of similar differences of opinion; but the great pitched battle over the question of surgical shock, between two rival leaders of American thought on this matter, did not come off owing to the absence of one of the combatants at the session devoted to this subject.

### Some Wonderful Operations.

In the surgical section there were many splendid contributions. The greatest public interest, perhaps, was evoked by the bone transplantation experiments and operations of Professor Albee, an American surgeon who was invited to demonstrate his methods on patients at the Royal National Orthopædic Hospital in Great Portland Street. This he did by performing his operation for tuberculosis of the spine (Pott's disease), which consists in grafting a piece of bone shaved off the surface of the shin bone or off a rib into the spinal column at the place where the disease has destroyed a portion of the vertebræ. Some fifty or sixty surgeons attended to see this operation, and as the operating theatre of the National Hospital was not built to hold this number, its resources were much strained.

An allied subject of great interest in which startling advances were reported was the restoration of mobility to stiff joints, fixed by the results of past inflammatory diseases. Dr. J. B. Murphy, of Chicago, and Professor Baer, of Baltimore, have done a great deal of work along these lines, which they described. The latter has succeeded in making fresh joint surfaces by grafting between the locked bones portions of mucous membrane taken from pigs, and has thus succeeded in making a stiff joint once more movable. Then there was the living patient shown by a well-known London surgeon, from whom the latter had four years ago removed the larynx and a considerable portion of the œsophagus for malignant disease. This patient has, of course, to breathe through a tracheotomy tube; and she has no direct natural channel left between the mouth and the stomach. She, therefore, has had a gastrotomy opening made into the latter organ; and an indiarubber tube leads from the throat down the front of the chest and into the stomach. Thus she is enabled to masticate her food, and to transfer it to the stomach through an artificial gullet. That this patient is alive and well for years after this operation speaks for itself.

